# Effects of chronic sleep deprivation on bone mass and bone metabolism in rats

**DOI:** 10.1186/s13018-016-0418-6

**Published:** 2016-08-02

**Authors:** Xiaowen Xu, Liang Wang, Liying Chen, Tianjiao Su, Yan Zhang, Tiantian Wang, Weifeng Ma, Fan Yang, Wujie Zhai, Yuanyuan Xie, Dan Li, Qiong Chen, Xuemei Fu, Yuanzheng Ma, Yan Zhang

**Affiliations:** 1 Center of Orthopedics, The 309th Hospital of PLA, Beijing, 100091 China; 2 Center for Systems Biomedical Sciences, University of Shanghai for Science and Technology, Shanghai, 200093 China

**Keywords:** Chronic sleep deprivation, Bone mineral density, Bone microstructure, Bone turnover, Vitamin D

## Abstract

**Background:**

This study aimed to assess the effects of chronic sleep deprivation (CSD) on bone mass and bone metabolism in rats.

**Methods:**

Twenty-four rats were randomly divided into CSD and control (CON) groups. Rats were subjected to CSD by using the modified multiple platform method (MMPM) to establish an animal model of CSD. Biochemical parameters such as levels of serum N-terminal propeptide of type I procollagen (PINP), N-terminal cross-linking telopeptide of type I collagen (NTX), growth hormone (GH), estradiol (E_2_), serum 25(OH)D, and calcium (Ca) were evaluated at 0, 1, 2, and 3 months. After 3 months, each fourth lumbar vertebra and the distal femoral metaphysis of the left extremity of rats were harvested for micro-computed tomography scans and histological analysis, respectively, after the rats were sacrificed under an overdose of pentobarbital sodium.

**Results:**

Compared with rats from the CON group, rats from the CSD group showed significant decreases in bone mineral density (BMD), bone volume over total volume, trabecular bone thickness, and trabecular bone number and significant increases in bone surface area over bone volume and trabecular bone separations (*P* < 0.05). Bone histomorphology studies showed that rats in the CSD group had decreased osteogenesis, impaired mineralization of newly formed bones, and deteriorative trabecular bone in the secondary spongiosa zone. In addition, they showed significantly decreased levels of serum PINP (1 month later) and NTX (3 months later) (*P* < 0.05). The serum 25(OH)D level of rats from the CSD group was lower than that of rats from the CON group after 1 month (*P* < 0.05).

**Conclusions:**

CSD markedly affects bone health by decreasing BMD and 25(OH)D, deteriorating the bone microarchitecture, and decreasing bone formation and bone resorption markers.

## Background

The American National Sleep Foundation recommends that healthy adults sleep for 7–9 h/day. However, an increasing number of people are having insufficient sleep, and approximately one third of adults report getting less than 6.5 h of sleep on each weeknight [1]. Owing to factors such as long working hours, internet and media use, stressful lifestyle, increased exposure to environmental light, and longer commuting time, more adults experience chronic sleep deprivation (CSD) in modern society [2]. Yang et al. reported that adolescents, who sleep approximately 4.86–5.62 h per day because of academic demands or stress and early school start times, also suffer from sleep deprivation [3]. Moreover, sleep problems increase with age. Several factors such as poor sleep hygiene, depression, use of medications, and comorbidities lead to disturbed sleep in elderly people, with 67 % of adults aged 65 years and older having at least one sleep-related complaint [4, 5]. Chronic sleep deprivation, owing to both short sleep duration and poor sleep quality, may contribute to problems in sustained attention, cognitive slowing [6], circadian rhythm disorders [7], cardiovascular disease [8], and increased risk of diabetes [9] and metabolic syndrome [10].

CSD has been reported to be associated with lower vitamin D concentrations [11]. Some clinical studies have shown that inadequate sleep duration and/or lower sleep efficiency is associated with lower vitamin D levels in elderly adults or older men [12–14]. Vitamin D affects intestinal calcium absorption and helps maintain the state of normocalcemia. It is an important hormone for bone health. A low serum 25(OH)D level is significantly associated with low bone mineral density (BMD). Therefore, some researchers have recently focused on the association between chronic sleep disturbances and BMD [15–21]. Several epidemiologic studies have shown that CSD leads to decreased BMD [15–18]. It has been reported that compared to individuals who sleep for 8 h, those who sleep for 6 h or less have significantly lower total and regional BMD [15]. Another clinical survey showed that patients with sleep disorders, particularly women and elderly people, have an increased risk of osteoporosis [16]. Similarly, young adults and children with poor sleep quality or short sleep duration have lower BMD and worse bone mass accrual, especially during periods of rapid growth [17, 18]. Several studies have suggested that CSD is associated with lower BMD; however, one study showed that sleeping for both less than and more than 8–9 h/day increases the risks of having osteoporosis [19]. In addition, some studies showed that the association between sleep duration and BMD was not significant [20, 21].

Previous clinical studies have failed to reach a consensus on the association between sleep duration and BMD, and have not indicated bone metabolic status and the related possible mechanisms associated with CSD. Owing to the difficulty in controlling diet and sleep behavior in humans through long periods of clinical research, animal laboratory studies are critical. Further animal laboratory studies are warranted to determine the relationship of short sleep duration with bone mass and bone metabolism. Thus, the present study aimed to explore the effects of long-term CSD on bone mass and bone metabolism in rats, and to further investigate the related possible mechanism. The modified multiple platform method (MMPM), as described in previous studies [22, 23], was selected to induce CSD. During the 3-month intervention of CSD, the lumbar spines of rats were subjected to micro-computed tomography (micro-CT) scans, the bone histomorphology of the left femur was analyzed, and serum levels of bone turnover markers, growth hormone (GH), estradiol (E_2_), serum 25(OH)D, and calcium (Ca) were evaluated.

## Methods

### Animals and experimental design

Twenty-four 5-month-old female Sprague-Dawley (SD) rats obtained from the Laboratory Animal Center of Military Medical Science Academy of PLA, Beijing, China, were randomly divided into chronic sleep deprivation (CSD) group (*n* = 12) and control (CON) group (*n* = 12). All animals were housed in six cages, with four rats in each cage, and kept under constant environmental conditions (22–24 °C, 12 h per day light/dark cycles). Food and water were offered ad libitum, except during the 12-h fasting period before the collection of blood samples. The body weight of the animals was measured once a week. After adapting to laboratory conditions for 30 min per day for 1 week, rats from the CSD group were placed on multiple small platforms, as described in the experimental procedure, while rats from the CON group were placed on a grid under the same conditions. The fasting blood samples were collected from the angular vein plexus between 08:00 and 10:00 h at 0, 1, 2, and 3 months, centrifuged at 1500×*g* for 10 min at 4 °C, and the serum sample was stored immediately at −80 °C for measurement of biochemical parameters. After 3 months, the rats were sacrificed under an overdose of pentobarbital sodium. The fourth lumbar vertebra [24] was harvested for micro-CT analysis, and the left femur was fixed in phosphate-buffered formaldehyde for histomorphological analysis. This study was carried out in strict accordance with the recommendations in the Guide for the Care and Use of Laboratory Animals of the National Institutes of Health. The animal use protocol has been reviewed and approved by the Ethics Committee of the 309th Hospital of PLA (number: 2015-13).

### Chronic sleep deprivation

The MMPM, illustrated elsewhere [23, 25], was selected to build the CSD model in this study. Rats from the CSD group were group housed (six rats in each arena) in modified multiple platform arenas during periods of sleep deprivation. The water tank (123 × 44 × 44 cm^3^), made of organic glass, contained 12 narrow circular platforms (6.5 cm in diameter) filled with water up to 1 cm of their upper surface. Thus, rats from the CSD group could move around freely inside the tank by jumping from one platform to another. When they reached the paradoxical phase of sleep, muscle atonia set in; their faces touched the water, and they awoke. Thus, the CSD models were built by depriving the rats of their paradoxical sleep. The housing condition for rats from the CON group was similar, but there was a grid floor covering on the narrow circular platforms. The grid floor was made of stainless steel, with the rods set 2 cm apart. This could provide a different environment for rats from the CON group, where they could lie down without falling into the water, while their tails may touch the water.

After a 1-week adaptation period, rats from the CSD group were sleep-deprived using the MMPM for 18 h (starting at 16:00 h) per day for 3 months. The animals were allowed to sleep in their individual home cages for the remaining time (6 h, 10:00–16:00 h). During a 3-month environmental period, the conditions in the water were kept constant (22–24 °C, 12 h per day light/dark cycles) and the water in the tank was changed daily. When all rats were housed in modified multiple platform arenas, chow pellets and water bottles were placed at the top of the tank, where the rats could freely access food and water.

### Micro-CT

The excised vertebrae (L4) were thawed at room temperature for 2 h before scanning. The bone microstructures of the fourth lumbar vertebrae were determined using a micro-CT with an Inveon Multimodality Imaging System (Siemens, Munich, Germany). The samples, placed in the sample holder of the scanner, were scanned along the longitudinal axis of the specimen, using the following scanning parameters: voltage of 80 kV, current of 500 μA, effective pixel size of 8.82 μm, and exposure time of 1500 ms in each of the 360 rotational steps [26]. The COBRA_Exxim system was used for image reconstruction. Parameters such as BMD, bone volume over total volume (BV/TV), bone surface area over bone volume (BS/BV), trabecular bone thickness (Tb.Th), trabecular bone number (Tb.N), and trabecular bone separations (Tb.Sp) were calculated using the Inveon Research Workplace (Siemens). Trabecular bone was determined by a fixed threshold. The interest region of lumbar vertebrae was isolated by hand-drawn contours based on approximately 200 consecutive slices (1.7 mm below the superior endplate of the lumbar vertebrae).

### Bone histology

The left femurs were decalcified in 10 % ethylenediaminetetraacetic acid (EDTA) (pH 7.4) for 2 months. They were then dehydrated using ethanol solutions and embedded in paraffin by using standard histological procedures. Sections of 4 μm were cut and stained with hematoxylin and eosin (H&E). Bone histology was evaluated using a microscope (Leica DM 2500). The images of chondrocyte zone at the growth plate and primary and secondary spongiosa at the distal femoral metaphysis of the extremities were captured.

### Serum biochemical parameters

Serum N-terminal propeptide of type I procollagen (PINP) was measured as bone-formation marker, while N-terminal cross-linking telopeptide of type I collagen (NTX) was selected as a bone resorption marker. Both PINP and NTX were measured using specific enzyme-linked immunosorbent assay (ELISA) kits (Beijing bioco Laibo Technology Co. Ltd., China). The intra- and inter-assay coefficients of variation for PINP were <7 and <8 %, respectively, and those for NTX were <6 and <10 %, respectively.

To explore the related possible mechanism, the changes in serum levels of GH and E_2_ as well as that of serum 25(OH)D, an important hormone linked with bone metabolism, were analyzed using ELISA kits (Beijing bioco Laibo Technology). In addition, serum calcium (Ca) levels were measured by a Beckman Coulter AU5800 clinical chemistry analyzer (USA).

### Statistical analysis

Data were presented as means ± standard deviation. The independent two-sample *t* test was used to identify significant differences between rats from the CSD and CON groups. All statistical comparisons were performed using SPSS (version 17.0). Statistical significance was set as *P* < 0.05.

## Results

### Body weight

**Fig. 1:**
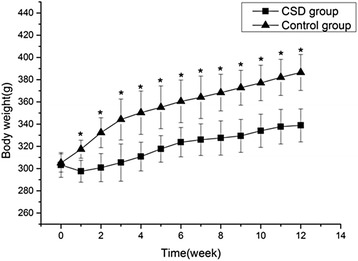
The body weight changes of the two groups. *CSD* chronic sleep deprivation, *CON* control. *P < 0.001 compared with the control group

The body weight of rats from both groups increased over time, but the increase in body weight of rats from the CSD group was significantly less than that of rats from the CON group after 1 week of sleep deprivation (Fig. 1, *P* < 0.001).

### Bone mass and bone microstructure analysis

**Fig. 2:**
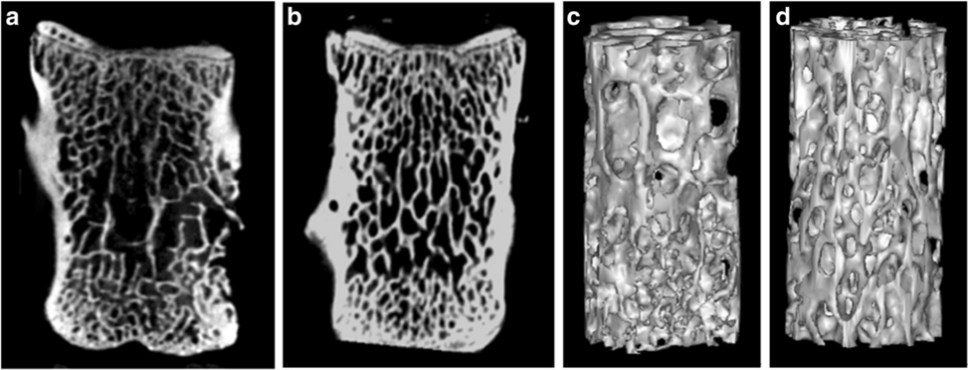
The representative micro-CT images of the fourth lumbar vertebrae in two groups. The two-dimensional coronal images (**a**, **b**) show that the CSD group (**a**) has increased disconnections and separation among trabecular bone network and reduced trabecular bone connection in the central region and under the endplate region compared to the CON group (**b**). The three-dimensional images of the trabecular bone microarchitecture (**c**, **d**) show that the CSD group (**c**) has thinner trabecular bone, reduced trabecular bone connection, and more irregular trabecular bone connection. And the CSD group (**c**) has increased disconnections and separation among the trabecular bone network compared to the CON group (**d**). *CSD* chronic sleep deprivation, *CON* control

The representative micro-CT images revealed that rats from the CSD group had fewer trabecular bone structures than those from the CON group (Fig. 2). The quantitative data of the fourth lumbar vertebrae were expressed as BMD, BV/TV, BS/BV, Tb.Th, Tb.N, and Tb.Sp (Table 1). The computed parameters showed significantly decreased BMD, BV/TV, Tb.Th, and Tb.N and significantly increased BS/BV and Tb.Sp in rats from the CSD group compared with those of the CON group (*P* < 0.05), suggesting that rats from the CSD group had lower bone mass and showed greater deterioration of the microarchitecture of the trabecular bone at the fourth lumbar vertebrae.

**Table 1:** Trabecular bone parameters of the fourth lumbar vertebrae measured by micro-CT

Parameters	CSD group	CON group	*P* value
BMD (mg/cm^3^)	1312.33 ± 8.94	1358.18 ± 10.15	0.000
BV/TV (%)	26.43 ± 3.51	35.00 ± 4.67	0.000
BS/BV (1/mm)	33.27 ± 2.49	30.51 ± 2.51	0.013
Tb.Th (mm)	0.061 ± 0.003	0.069 ± 0.005	0.000
Tb.N (1/mm)	4.45 ± 0.31	4.95 ± 0.34	0.001
Tp.Sp (mm)	0.20 ± 0.02	0.17 ± 0.03	0.008

Data were presented as means ± SD. *CSD* chronic sleep deprivation, *CON* control, *BMD* bone mineral density, *BV/TV* bone volume/total volume, *BS/BV* bone surface area/bone volume, *Tb.Th* trabecular bone thicknesses; *Tb.N* trabecular bone numbers, *Tb.Sp* trabecular bone separation

### Bone histomorphology

**Fig. 3:**
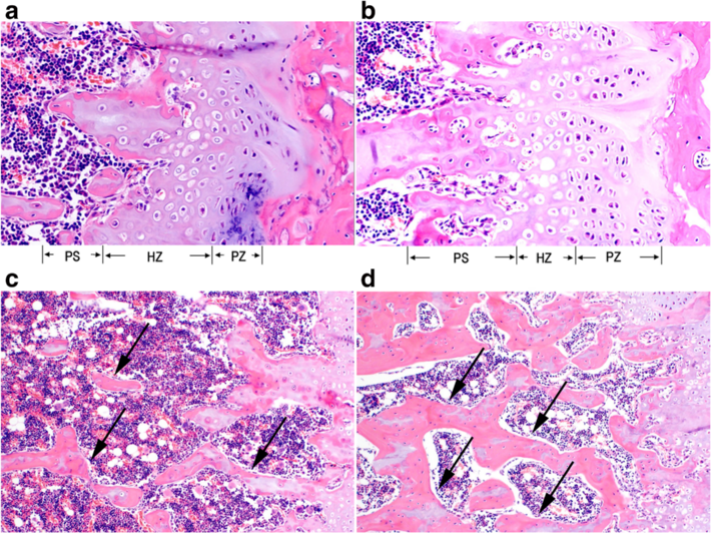
The representative histological images of the extremity distal femoral metaphysis in two groups. The chondrocyte zone images at growth plate (**a**, **b**) show that the CSD group (**a**) has a shorter proliferative zone (PZ), increased hypertrophic zone (HZ) of chondrocytes, and decreased osteoid content of the primary spongiosa (PS) zone. The secondary spongiosa zone images (**c**, **d**) show that CSD group (**c**) has increased disconnections and separation among the trabecular bone network and a decreased trabecular bone mass compared to the CON group (**d**). The *arrows* point the trabecular bone. Magnification of **a** and **b**, ×200; magnification of **c** and **d**, ×100. *PZ* proliferative zone, *HZ* hypertrophic zone, *PS* primary spongiosa zone, *CSD* chronic sleep deprivation, *CON* control

The representative histological images of the distal femoral metaphysis of the extremities revealed remarkable bone abnormalities in rats from the CSD group (Fig. 3). The proliferative zone of rats from the CSD group was shorter than that of rats from the CON group, suggesting decreased osteogenesis in rats from the CSD group. In addition, rats from the CSD group had an increased hypertrophic zone of chondrocytes and a decreased osteoid content of the primary spongiosa zone, indicating impaired mineralization of the newly formed bones (Fig. 3a, b). In the secondary spongiosa zone, rats from the CSD group showed increased disconnections and separation among the trabecular bone network and a decreased trabecular bone mass (Fig. 3c, d).

### Serum biochemical parameters

**Fig. 4:**
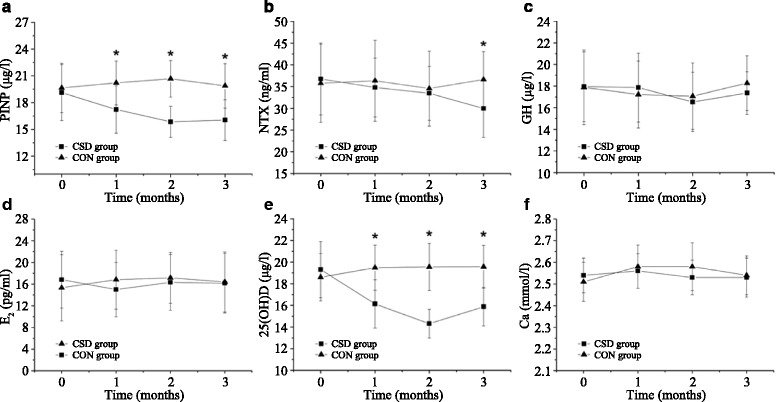
The changes of the serum biochemical parameters during experimental period. **a** N-terminal propeptide of type I procollagen (PINP). **b** N-terminal cross-linking telopeptide of type I collagen (NTX). **c** Growth hormone (GH). **d** Estradiol (E2). **e** 25(OH)D. **f** Calcium (Ca). *CSD* chronic sleep deprivation, *CON* control. *P < 0.05 compared with the control group

The changes in the serum levels of PINP and NTX are shown in Fig. 4a, b. Compared to rats from the CON group, rats from the CSD group showed significantly decreased levels of serum PINP after 1 month of CSD (*P* < 0.05), whereas the levels of serum NTX in rats from the CSD group significantly decreased after 3 months of CSD (*P* < 0.05).

The serum levels of GH, E_2_, and Ca showed no significant changes during the experiment, and the differences between their levels in rats from the CSD and CON groups were not significant (Fig. 4c, d, f; *P* > 0.05). The changes in the serum levels of 25(OH)D are shown in Fig. 4e. Compared to rats from the CON group, those from the CSD group showed significantly decreased levels of serum 25(OH)D after 1 month of CSD (*P* < 0.05).

## Discussion

In the present study, we observed that CSD resulted in lower bone mass and deterioration of bone microarchitecture, including significant decreases in BMD, BV/TV, Tb.Th, and Tb.N, and significant increases in BS/BV and Tb.Sp. Bone histomorphology studies showed that rats from the CSD group had decreased osteogenesis, impaired mineralization of the newly formed bones, and deteriorative trabecular bone in the secondary spongiosa zone. Both bone formation and resorption marker levels (PINP and NTX) were significantly decreased, suggesting a low bone turnover after 3 months of CSD, which could be attributed to the decreased serum levels of 25(OH)D observed after 1 month of CSD. However, the serum levels of GH, E_2_, and Ca showed no significant changes during the experiment, and the differences between their levels in rats from the CSD and CON groups were not significant. In addition, the body weights of rats from both groups increased over time; however, CSD resulted in attenuation of weight gain after 1 week of sleep deprivation.

Although several clinical studies showed an association between sleep duration and BMD, the conclusions have not reached a consensus. Specker et al. [27] reported that compared to women who got adequate sleep (>6.5–10 h/night), sleep-deprived women (<6.5 h/night) had lower cortical volumetric BMD after adjustment for potential covariates, such as age, weight, height, physical activity, and intakes of fat, calcium, and vitamin D. Fu et al. [15] reported that compared to individuals who slept for 8 h, those who slept for a shorter duration of 6 h or less had significantly lower total and regional BMD. Similar results were reported in other studies on children and young adults; those with poorer sleep quality or shorter sleep duration also had lower BMD and worse bone mass accrual, especially during periods of rapid growth [17, 18]. In contrast, Nakagi et al. [20] reported that sleep duration was not significantly correlated with BMD. Another study also did not support the association between shorter sleep duration and lower BMD levels in older adults. The study indicated that men, but not women, who slept for ≥9 h/day had significantly lower femoral neck BMD than those who slept for 8 h/day after adjusting for age, education level, smoking, physical activity, depressive symptomatology, comorbidity, and serum vitamin D concentration; however, the association was weak and showed no significant difference after further adjustment for urinary cortisol and serum inflammation biomarkers [28]. Another large-scale cross-sectional study suggested that both shorter (7–8 h/day) and longer (9–10 h/day and ≥10 h) sleep duration tends to increase the risk of osteoporosis in postmenopausal women [19]. They provided two possible reasons for the effect of longer sleep on bone. One explanation was that the increase in total sleep duration during the day would reduce the time that could be spent doing physical exercise or labor. Another explanation was that the longer sleep may be a consequence associated with the influence of chronic disease. However, an animal laboratory study on the relationship between shorter sleep duration and BMD showed that new bone formation in sleep-restricted rats decreased without reduction in bone resorption, leading to osteopenia [29]. This finding is consistent with that of the present study, which showed that rats with CSD had lower bone mass. Nevertheless, both bone formation and resorption in rats significantly decreased in our study; further studies are warranted to confirm the effects of bone metabolism. A previous study reported that the histomorphometric determinations of bone formation, such as growth plate or cortical bone thicknesses observed in pathologic pictures, showed no statistically significant difference between sleep-restricted rats and controls. In contrast, we used micro-CT, which has been acknowledged as an effective technique for investigating the microarchitecture of bone for excised applications [30], to evaluate the microarchitecture of each fourth lumbar vertebra. We found remarkably deteriorating bone microarchitecture in rats from the CSD group compared with controls, including significant decreases in BV/TV, Tb.Th, and Tb.N and significant increases in BS/BV and Tb.Sp. Bone histomorphology of the distal femoral metaphysis of the extremity demonstrated that rats from the CSD group showed decreased osteogenesis, impaired mineralization of the newly formed bones, and deteriorative trabecular bone in the secondary spongiosa zone.

Circadian rhythm disruption was a routine consequence of CSD. It was reported that CSD may lead to GH deficit [31]. GH is an important regulator of postnatal growth and bone mass. It has been reported that GH can improve bone mass and increase the mechanical strength of bone in glucocorticoid-induced/aged ovariectomized osteopenic and normal rodents [32, 33]. Grubbe et al. [34] reported a similar result that GH mitigates loss of periosteal bone formation and muscle mass in disuse osteopenic rats. Further, Iglesias et al. reported that GH deficiency, established by hypophysectomy, results in cessation of bone growth and a decrease in cancellous bone mass [35]. However, our study did not find any significant difference of GH between rats from the CSD and CON groups. This could be attributed to fluctuations in the serum GH level during the long-term collection of fasting blood samples in our experimental design. Owing to the relatively small sample size in our experiment, further large-sample experiments are required. Sex hormone is another important regulator of bone mass. Estrogen deficiency could increase the risk of osteoporosis in postmenopausal women. It has been reported that serum estrogen level of paradoxical sleep-deprived rats was lower than that of normal rats [36]. Nonetheless, as the growth hormone, the difference of E_2_ between rats from the CSD and CON groups was not significant in the present study.

Vitamin D is an important hormone for bone health. It affects intestinal calcium absorption and maintains the state of normocalcemia. Low serum 25(OH)D level was reported to be significantly correlated with low BMD and increased risk of non-vertebral and hip fracture [37–39]. Vitamin D deficiency in children causes rickets, while in adults, it may lead to osteomalacia. Limited clinical studies have shown the association between vitamin D level, and sleep duration and quality. Kim et al. [13] reported that inadequate sleep duration is associated with lower vitamin D levels in elderly people. Massa et al. [14] reported that low serum 25(OH)D level in older men is related with poorer sleep, which is a combination of short sleep duration and low sleep efficiency. In contrast, Gunduz et al. [40] reported that despite the prevalence of insufficient vitamin D and poor sleep quality in pregnant women, low levels of vitamin D are not associated with poor sleep quality. However, in the present study, rats from the CSD group showed significantly decreased levels of serum 25(OH)D after 1 month of CSD. The low serum 25(OH)D level in these rats may be an important factor responsible for the lower bone mass, deteriorated bone microarchitecture, and low bone turnover.

We also found that the body weights of rats from both groups increased over time; however, CSD resulted in attenuation of weight gain after 1 week of sleep deprivation. A similar result was observed in previous studies, where sleep-deprived rats lost 15 % of their body weight, whereas their food intake increased 2- to 3-fold [29, 41]. This negative energy balance may have an adverse effect on bone formation. It has been reported that the negative energy balance induced an inverse effect on osteocalcin concentrations [42]. Yamamoto et al. [43] found that hypermetabolism induced by thyroid hormone administration results in osteopenia. In the present study, rats from the CSD group showed decreased bone mass, suggesting the involvement of negative energy balance in the effect of CSD on bone mass. Further studies are warranted to explore the possible mechanism. In addition, in this study, the serum levels of Ca showed no significant changes during the experiment, and the differences in Ca levels between rats from the CSD and CON groups were not significant. This could be attributed to the strict regulation of serum Ca level by using multiple approaches to maintain steady concentration with little fluctuation.

Some studies have reported that chronic poor sleep may contribute to chronic low-grade inflammation [44, 45]. This inflammation is associated with an increased risk for osteoporosis. Smith et al. developed an in vivo model of bone loss induced by chronic systemic inflammation by using SD rats, and observed upregulation of proinflammatory mediators such as cyclooxygenase (COX)-2, interleukin (IL)-1, and tumor necrosis factor (TNF)-alpha in the metaphyseal region [46]. Jurado et al. reported that IL-1beta treatment could increase the level of OPG mRNA in primary human osteoblastic cell [47]. D’Amelio et al. found that estrogen deficiency enhances the production of the pro-osteoclastogenetic cytokine TNF-alpha, and observed that T cells and monocytes from women with osteoporosis exhibit a higher production of TNF-alpha [48]. Oxidative stress, caused by increased reactive oxygen species, has a negative age-related effect on BMD in rats and could accelerate bone deterioration in aged rats [49]. The possible reason is that it could inhibit the proliferation and differentiation of rat osteoblasts. These studies indicate the involvement of some inflammatory cytokines in the effect of CSD on bone health. However, we did not detect inflammatory cytokines in this study. Further studies are warranted to evaluate the role of inflammatory cytokines in the effect of CSD on bone health.

Some hypotheses were proposed to explain the effects of CSD on bone mass and bone metabolism. It was reported that the skeleton may be a ductless gland that could produce a hormone responding to environmental influences of metabolism and energy requirements. The bone hormone, in cooperation with other hormones, may regulate phosphate metabolism and influence skeletal mineralization [50, 51]. Meanwhile, the CSD rat model of MMPM could disturb the endocrine system. The bone hormone may be disturbed by CSD, influencing the bone mass and bone metabolism. In addition, the sleep loss and sleep disturbances could accelerate the senescence process [52, 53], which is an important risk factor for osteoporosis with low bone transformation. Thus, CSD may lead to low bone transformation and reduced bone mass by accelerating the senescence process. The results of this study were consistent with the proposed hypotheses.

Some limitations of the present study deserve attention. First, despite evaluating the effect of a short sleep duration of 6 h on bone mass and bone metabolism, the extent of restrictive sleep durations was not considered in the present study. Several groups of different sleep durations should be established in future studies. Second, the changes in bone mass, bone microarchitecture, and serum biochemical parameters after the recovery of sleep for several months were not analyzed. It is unclear whether the effect of CSD on bone health is a reversible process. Third, since the serum levels of GH, E_2_, and Ca were found to fluctuate at different times within 24 h, the fasting blood samples, collected between 08:00 and 10:00 h, could not entirely represent the status of GH, E_2_, and Ca in rats. A more rational experimental design should be applied in future studies. Finally, the sample size of this study was relatively small, whereas the standard deviation was a little large; thus, the results should be explained with caution, and a larger sample should be used in future studies.

## Conclusions

In conclusion, our data reveal that CSD markedly affects bone mass and bone metabolism, by lowering BMD, deteriorating bone microarchitecture, and decreasing bone formation and resorption markers. Serum levels of 25(OH)D decreased after 1 month of chronic sleep deprivation, whereas the serum levels of GH, E_2_, and Ca showed no significant changes during the experiment. It is recommended that a good sleep pattern be maintained for optimal bone health. Further studies are warranted to confirm the association between CSD and bone health, and to explain the potential mechanism underlying the effects of CSD on bone health.

## Abbreviations

BMD, bone mineral density; Ca, calcium; CON, control; CSD, chronic sleep deprivation; GH, growth hormone; micro-CT, micro-computed tomography; MMPM, modified multiple platform method
